# Aequorin-based luminescence imaging reveals differential calcium signalling responses to salt and reactive oxygen species in rice roots

**DOI:** 10.1093/jxb/erv043

**Published:** 2015-03-09

**Authors:** Yanyan Zhang, Yifeng Wang, Jemma L. Taylor, Zhonghao Jiang, Shu Zhang, Fengling Mei, Yunrong Wu, Ping Wu, Jun Ni

**Affiliations:** ^1^College of Life and Environmental Sciences, Hangzhou Normal University, Hangzhou 310036, China; ^2^State Key Laboratory of Plant Physiology and Biochemistry, College of Life Science, Zhejiang University, Hangzhou 310058, China; ^3^School of Life Sciences, Gibbet Hill Campus, University of Warwick, Coventry CV4 7AL, United Kingdom

**Keywords:** Aequorin, calcium, *Oryza sativa*, reactive oxygen species, rice, root, salt.

## Abstract

It is well established that both salt and reactive oxygen species (ROS) stresses are able to increase the concentration of cytosolic free Ca^2+^ ([Ca^2+^]_i_), which is caused by the flux of calcium (Ca^2+^). However, the differences between these two processes are largely unknown. Here, we introduced recombinant aequorin into rice (*Oryza sativa*) and examined the change in [Ca^2+^]_i_ in response to salt and ROS stresses. The transgenic rice harbouring aequorin showed strong luminescence in roots when treated with exogenous Ca^2+^. Considering the histological differences in roots between rice and *Arabidopsis*, we reappraised the discharging solution, and suggested that the percentage of ethanol should be 25%. Different concentrations of NaCl induced immediate [Ca^2+^]_i_ spikes with the same durations and phases. In contrast, H_2_O_2_ induced delayed [Ca^2+^]_i_ spikes with different peaks according to the concentrations of H_2_O_2_. According to the Ca^2+^ inhibitor research, we also showed that the sources of Ca^2+^ induced by NaCl and H_2_O_2_ are different. Furthermore, we evaluated the contribution of [Ca^2+^]_i_ responses in the NaCl- and H_2_O_2_-induced gene expressions respectively, and present a Ca^2+^- and H_2_O_2_-mediated molecular signalling model for the initial response to NaCl in rice.

## Highlight

Based on the aequorin system, we describe the establishment of a calcium reporter line in rice and test the responses of the reporter towards salt and oxidative stress.

## Introduction

Rice (*Oryza sativa* L.) is the staple food for more than half of the world’s population. Salinity is one of the most common abiotic stresses encountered by rice, which is classified as a salt-sensitive crop in early stages of development, and limits its productivity (Lutts *et al.*, 1995; Todaka *et al.*, 2012). To improve the rice yield under saline conditions, it is important to understand the molecular mechanisms involved in how rice responds to salt stress (Kumar *et al.*, 2013).

Many studies have been carried out to dissect the genetic and molecular mechanisms of how plants respond to salt stress. A well-defined pathway is the Salt Overly Sensitive (SOS) signalling pathway, which comprises SOS3, SOS2 and SOS1 (Zhu, 2000), and is required to mediate the highly complex regulatory networks involved in plant response to salinity (Ji *et al.*, 2013). Importantly, the SOS signal transduction cascade is activated by a calcium (Ca^2+^) spike, which is caused by the flux of Ca^2+^. This salt stress triggered increase of cytosolic free Ca^2+^ ([Ca^2+^]_i_) is considered to be the first recorded response to salt stress (Knight *et al.*, 1997; Tracy *et al.*, 2008). Ca^2+^ is an essential second messenger in the sophisticated network of plant signalling pathways responding to a large array of external stimuli, including salt stress (Hetherington and Brownlee, 2004; Pandey *et al.*, 2004; Dodd *et al.*, 2010). The Ca^2+^ channels and transporters activated by these stimuli form specific Ca^2+^ signatures, and these changes in Ca^2+^ signatures are transmitted by protein sensors that preferably bind Ca^2+^. The binding of Ca^2+^ results in conformational changes which modulate their activity or their ability to interact with other proteins, and activate the expression of downstream salt response genes through a Ca^2+^ signalling cascade (Rentel and Knight, 2004; Dodd *et al.*, 2010; Kudla *et al.*, 2010; Batistic and Kudla, 2012).

Furthermore, salt stress also increases the level of reactive oxygen species (ROS) predominantly represented by H_2_O_2_ (Bienert *et al.*, 2006; Hong *et al.*, 2009; Miller *et al.*, 2010). Although toxic by nature, ROS are now considered as important signalling molecules in many biological processes, including biotic and abiotic stress tolerance (Mittler *et al.*, 2011; Schippers *et al.*, 2012). In *Arabidopsis*, Respiratory Burst Oxidase Homolog F (RBOHF, an NADPH oxidase catalysing ROS production) is required for shoot sodium homeostasis during salt stress (Jiang *et al.*, 2012). Furthermore, the RBOHF-dependent salinity-induced ROS accumulation is regulated by protein phosphorylation in a Ca^2+^-dependent manner (Drerup *et al.*, 2013). Interestingly, ROS have also been shown to trigger an increase of [Ca^2+^]_i_ (Mori and Schroeder, 2004). Previous limited evidence implied that NaCl-gated Ca^2+^ channels and H_2_O_2_-gated Ca^2+^ channels may differ (Jiang *et al.*, 2013). However, the different mechanisms between NaCl- and H_2_O_2_-induced [Ca^2+^]_i_ changes are yet to be explored. Also, SALT-RESPONSIVE ERF1 (SERF1) is reported to function as a central hub to regulate ROS-dependent signalling during the initial response to salt stress in rice (Schmidt *et al.*, 2013). Therefore, it is attractive to investigate the relationship among Ca^2+^, H_2_O_2_ and SERF1 in the early salt-stress signalling cascade.

Genetically encoded fluorescent Ca^2+^ probes are useful tools to non-invasively describe Ca^2+^ signatures in plants (Monshausen, 2012). The fluorescence resonance energy transfer (FRET)-based probes yellow cameleon YC2.1 (Miyawaki *et al.*, 1999) and the improved YC3.6 (Nagai *et al.*, 2004) are currently used to monitor the [Ca^2+^]_i_ in the cytoplasm. With high quantum yield, FRET-based probes are suitable to measure the Ca^2+^ signatures in the cellular or subcellular resolutions (Monshausen, 2012). Aequorin, a photoprotein derived from the luminescent jellyfish *Aequoria victoria*, reacts specifically with Ca^2+^ and emits blue light at ~460nm (Shimomura *et al.*, 1963). Although the aequorin-based probe gives a low quantum yield, it is more suitable for cell population or whole plant measurement of [Ca^2+^]_i_ (Monshausen, 2012). Since the transformation of recombinant aequorin in plant systems (Knight *et al.*, 1991), it has proved to be a useful tool for non-invasive investigation of Ca^2+^-mediated signalling in response to various stresses in whole seedlings (Zhu *et al.*, 2013). Specific stimuli can trigger unique Ca^2+^ signatures, which are decoded subsequently by intracellular Ca^2+^ sensors, leading to the activation of downstream events (Luan, 2009). Kurusu *et al.* (2011) established a transgenic rice cell line expressing apoaequorin, and characterized the regulation mechanism of microbe-associated molecular pattern-induced [Ca^2+^]_i_ transients. In spite of the progress achieved by rice cell lines, stable transgenic rice expressing apoaequorin is needed to investigate the changes of [Ca^2+^]_i_ that appear in response to various environmental stimuli.

In this paper, we introduced recombinant aequorin, as a reporter of [Ca^2+^]_i_, into rice. Transgenic rice harbouring aequorin showed strong luminescence in roots when treated with exogenous Ca^2+^. We also showed that NaCl and H_2_O_2_ treatments induce different [Ca^2+^]_i_ spikes, and may employ different Ca^2+^ channels. Furthermore, we present a Ca^2+^- and H_2_O_2_-mediated molecular signalling model for the initial response to NaCl in rice.

## Materials and methods

### Vector construction and transformation of rice

In order to improve the aequorin expression vector for transgenic research in rice, the coding region of apoaequorin in *pMAQ2* (Knight *et al.*, 1991) was transferred to *35S-pCAMBIA1301* (Zhou *et al.*, 2008) through *Xba*I and *Pst*I. Plasmids were introduced into *Agrobacterium tumefaciens* EHA105 by electroporation. Rice transformation was performed by the *Agrobacterium*-mediated method, as previously described (Chen *et al.*, 2003).

### Plant materials and growth conditions

Rice (*Oryza sativa* L. cv. Nipponbare) seeds were sterilized with 75% ethanol and planted in a square plate containing half-strength Murashige and Skoog salts (MS; Gibco), and 1.5% (w/v) agar (Becton Dickinson). Seedlings were grown vertically in the growth chamber conditioned with 16h of light at 28°C and 8h of dark at 22°C for five days. The seedlings were then sprayed with coelenterazine for reconstitution of aequorin before subsequent experiments began.

### Root cell death detection

A root cell death assay was performed as previously described (Qin *et al.*, 2013). Roots of five-day-old seedlings were submerged in different concentrations of NaCl solution for 30 s and then stained with 1% Evans blue solution for 10min, washed by distilled water for 2h, and then photographed.

### Southern-blot analysis of transgenic rice

Genomic DNA of transgenic rice was isolated following the instructions of a Plant Genomic DNA Kit (TIANGEN) and the purified DNA was digested with restriction enzyme *Eco*RI. 2 µg of digested DNA was separated on 0.8% agarose gel. After electrophoresis, the digested DNA was transferred to Hybond-N+ nylon membrane (Amersham Pharmacia) and hybridized with a ^32^P-dCTP-labelled hygromycin-resistant gene probe. The blots were washed at 65°C under stringent conditions and analysed using Typhoon-8600. The primers used to amplify the probe are listed in Supplementary Table S1.

### RT-PCR analysis

For the examination of apoaequorin expression in different tissues, the shoot, shoot base and root of five-day-old transgenic rice seedlings were selected, and the PCR was conducted with 28 cycles for both apoaequorin and *OsACTIN*. For the examination of NaCl and H_2_O_2_ induced gene expression, the roots of five-day-old rice seedlings were selected. Ca^2+^ channel blocker pre-treatment was performed by 1mM LaCl_3_ treatment for 30min. NaCl treatment was performed by 0.15M NaCl treatment for 1h. H_2_O_2_ treatment was performed by 1mM H_2_O_2_ treatment for 1h. The relative expression levels were calculated according to the 2^-∆∆Ct^ method (Livak and Schmittgen, 2001). Each experiment was carried out with three independent biological replications. For RT experiments, 5 μg of total RNA was denatured at 65°C for 5min followed by quick chill on ice in a 14 μl reaction containing 1 μl oligo (dT)_12–18_ (500 μg ml^-1^) primer, and 1μl of 10mM dNTP mixture (10mM each dATP, dGTP, dCTP, and dTTP at neutral pH). After addition of 4 μl 5× reaction buffer (Promega), the reaction was incubated at 37°C for 2min, and 1 μl (200 units) of M-MLV RT^a^ (Promega) was added to the reaction and incubated at 42°C for another 50min. For inactivation, the reaction was heated at 70°C for 15min. The primers are listed in Supplementary Table S1.

### Aequorin reconstitution and luminescence imaging

Seedlings were grown on half-strength MS medium for five days. Reconstitution of aequorin was performed *in vivo* by spraying seedlings with 10 µM coelenterazine and followed by incubation at 21°C in the dark for 12–16h. For surfactant treatment, 0.01% or 0.1% of silwet L-77 (Sigma) was added to the coelenterazine solution. For Ca^2+^ inhibitor treatments, rice roots were treated with different concentrations of GdCl_3_, LaCl_3_, neomycin and thapsigargin, respectively for 30min before 0.25M NaCl and 1mM H_2_O_2_ treatment. Treatments and aequorin luminescence imaging were performed at room temperature using a ChemiPro HT system as described previously (Jiang *et al.*, 2013). The recording was started about 5 s prior to treatment and luminescence images were acquired for 3min. For the analysis of time courses of increase in [Ca^2+^]_i_, each exposure time was 30 s and the images were taken continuously for several minutes. To avoid the interference of chloroplast auto-fluorescence signal in the aequorin luminescence imaging, all the treatments were performed in the complete darkness. To record the chloroplast auto-fluorescence signal, seedlings were first exposed to strong light for 1min. After that, the light was turned off and the chloroplast auto-fluorescence was recorded. WinView/32 and Meta Morph 7.7 were used to analyse recorded luminescence images.

## Results

### Production and characterization of transgenic rice expressing apoaequorin

**Fig. 1.**
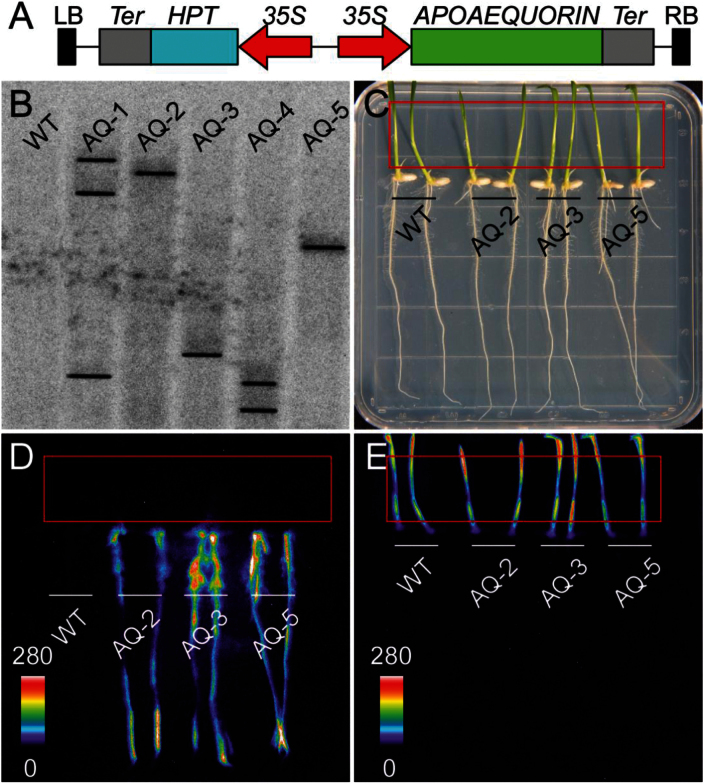
Transgenic rice harbouring aequorin showed strong and diverse luminescence in roots. (A) The construction of apoaequorin expression vector for transgenic research in rice. (B) Southern-blot analysis of five independent lines (AQ-1 to AQ-5) of transgenic rice. (C-E) Ca^2+^-treated seedlings showed strong and diverse luminescence exclusively in roots. (C) Bright-field image. (D) Pseudocolour image of aequorin luminescence in roots. (E) Pseudocolour image of chloroplast auto-fluorescence. Red rectangles indicate the places of shoots, and there is no aequorin luminescence signal in shoots shown in (D). The relationship between luminescence intensity and the pseudocolour images are scaled by pseudocolour bars.

In order to monitor [Ca^2+^]_i_ responses in rice, we developed transgenic rice over-expressing apoaequorin under the control of cauliflower mosaic virus (CMV) 35S promoter (Fig. 1A). Three independent transgenic lines (AQ-2, AQ-3 and AQ-5) harbouring one copy of apoaequorin were selected by Southern blot analysis, and the homozygous T_3_ generations of these lines were used for subsequent experiments (Fig. 1B). In addition, the heterologous expression of apoaequorin had no effects on the growth and life cycle of transgenic rice (data not shown). After the reconstitution of aequorin by spraying seedlings with coelenterazine, the aequorin luminescence of these seedlings were recorded using a photo-counting camera by treating plants with exogenous Ca^2+^ (see *Materials and methods* for detail). Ca^2+^-treated seedlings showed strong and diverse luminescence in roots, and AQ-3 with the strongest luminescence was selected for further analysis (Fig. 1D). To our surprise, the aequorin-based luminescence signal was only observed in roots and we failed to detect any signal in shoots when treated with Ca^2+^ (Fig. 1D compared to bright-field in Fig. 1C and chloroplast auto-fluorescence in Fig. 1E). To confirm the expression of apoaequorin in the whole plant, we extracted RNA from different tissues of transgenic seedlings, and the expression of apoaequorin was examined using reverse transcription-polymerase chain reaction (RT-PCR). The results showed that apoaequorin is expressed in all the selected tissues (Supplementary Fig. S1A). It is likely that the leaf wax prevents the permeating of coelenterazine (Supplementary Fig. S1B). To test this hypothesis, we added surfactant (Silwet L-77, Sigma) while spraying coelenterazine. Both luminescence signals in roots and dotted signals in shoots were observed (Supplementary Fig. S1C–H). These results showed that transgenic rice expressing apoaequorin was able to reflect the [Ca^2+^]_i_ level in rice roots.

### The optimization of discharging solution for luminescence imaging in rice

The discharging solution is used to estimate the amount of remaining aequorin in the calibration and is important to calculate the Ca^2+^ concentration based on the luminescence intensity (Knight *et al.*, 1996). Considering the histological differences in roots between rice and *Arabidopsis* (Rebouillat *et al.*, 2009), we re-examined the percentage of ethanol in the discharging solution for the rice experiment. We tested a series of discharging solutions with different percentages of ethanol from 0% to 50%. Low percentages of ethanol (below 15%) had no significant difference in the luminescence imaging compared with 0% ethanol. Interestingly, the average luminescence intensity of plants increased by about one fold when treated with discharging solution containing 20% ethanol compared with that treated with a low concentration of ethanol (Supplementary Fig. S2). In spite of the leap from 15% to 20%, even higher percentages of ethanol in the discharging solution had little effect in the luminescence intensity of rice, indicating the saturation of discharged aequorin. Based on these results, we suggested that in contrast with 10% ethanol which is normally used in *Arabidopsis* (Yuan *et al.*, 2014), the percentage of ethanol in the discharging solution for rice should be 25%.

### NaCl induced an immediate [Ca^2+^]_i_ spike in rice roots

**Fig. 2.**
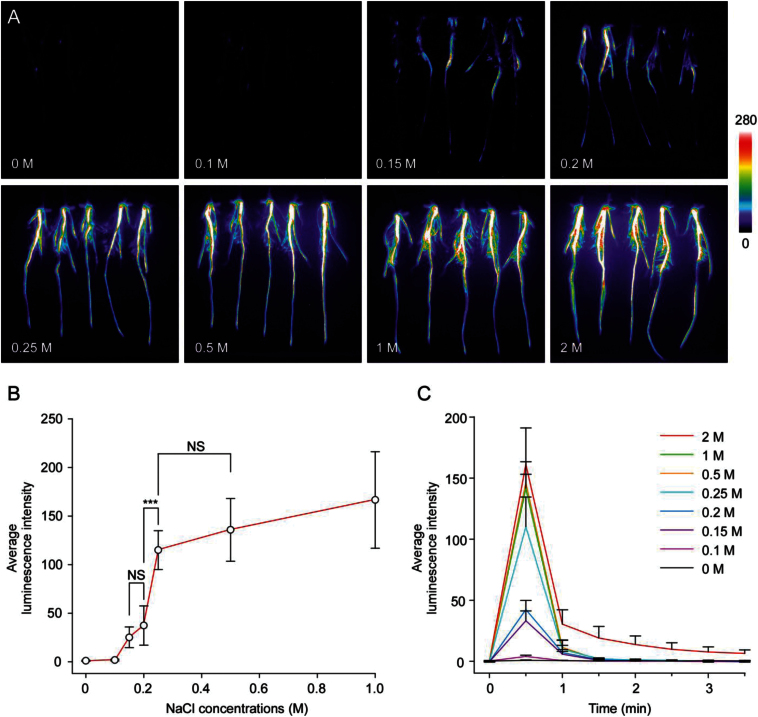
Aequorin-based luminescence in roots under NaCl treatments. (A) Pseudocolour images of aequorin luminescence in roots treated with different concentrations of NaCl. The relationship between luminescence intensity and the pseudocolour images are scaled by a pseudocolour bar. (B) The line chart of luminescence signal intensity of every treatment. (C) The time courses of [Ca^2+^]_i_ changes induced by different concentrations of NaCl in rice roots. Data for independent experiments are shown (mean±sd; *n*=10; *** *P*<0.001; NS, not significant *P*>0.05; Student’s *t*-test).

In order to investigate the [Ca^2+^]_i_ changes in response to salt stress in rice roots, we examined the aequorin-based luminescence under various concentrations of NaCl treatments. The intensity of [Ca^2+^]_i_-dependent luminescence signals relied on the strength of salt stimuli (Fig. 2A). Detailed analysis showed that 0.1M (or less) of NaCl failed to induce visible [Ca^2+^]_i_-dependent luminescence signals, while 0.15M NaCl successfully induced a visible concentration of luminescence signals. 0.25M NaCl had a more obvious effect than 0.2M, and was very similar to 0.5M NaCl treatments (Fig. 2A). To further investigate the salt concentration-dependent increase of [Ca^2+^]_i_ in rice roots, we calculated the average luminescence intensity of rice roots in response to different concentrations of NaCl treatments. As shown in Fig. 2B, a rapid increase of luminescence intensity in response to NaCl treatment occurred within a narrow range of NaCl concentrations (0.1M to 0.25M). NaCl treatments below or above this region had minor effect on the [Ca^2+^]_i_ responses in rice roots (Fig. 2B).

To investigate the time courses of [Ca^2+^]_i_ responses induced by different concentrations of NaCl in rice roots, the average luminescence intensity of continuous images with exposure time of 30 s were analysed and a comparison of basic parameters (amplitudes, durations and phases) of [Ca^2+^]_i_ responses were made. As shown in Fig. 2C, a strong luminescence signal was detected in the first image, which collected the luminescence signal within the first 30 s. However, almost no luminescence signal was detected after the first 30 s. This indicated that salt stress immediately induced a sharp spike of [Ca^2+^]_i_ within 30 s, which quickly declined to the basal level after the spike. The amplitudes of luminescence signals varied according to different concentrations of NaCl, while both the durations and phases were the same. Detailed analysis showed that 0.1M NaCl had little effect on the induction of [Ca^2+^]_i_, while 0.15M NaCl was able to induce an obvious spike of [Ca^2+^]_i_. The effect of 0.2M NaCl was not significantly different in the increase of amplitude of [Ca^2+^]_i_ increase compared with 0.15M. Interestingly, increasing the concentration of NaCl to 0.25M dramatically increased the amplitude of [Ca^2+^]_i_ by about two fold, but concentrations higher than 0.25M NaCl had little effect on the increasing of amplitude compared with 0.25M (Fig. 2C). These results were similar to the curves shown in Fig. 2B. It is worth noting that the spike of [Ca^2+^]_i_ failed to decline to the basal level after the induction by 2M NaCl treatment (Fig. 2C). This indicated a destruction of calcium transport systems, which are responsible for maintaining low [Ca^2+^]_i_, and crucial to the living cells. As expected, Evans blue staining revealed that treatment with a high concentration of NaCl resulted in serious cell death in the rice roots (Supplementary Fig. S3).

### H_2_O_2_ induced a delayed [Ca^2+^]_i_ spike in rice roots

In order to investigate the [Ca^2+^]_i_ changes in response to ROS stress in rice roots, we examined the aequorin-based luminescence after the application of H_2_O_2_. We collected the luminescence signals 45 times at one minute intervals after treatment with H_2_O_2_. We found clear luminescence signals in the first and second minute. Interestingly, after the weak luminescence signals in the third minute, we failed to collect any additional signals over the remaining 42min (Supplementary Fig. S4). This was different from that in *Arabidopsis*, which was reported to have a second peak 5–20min after the application of H_2_O_2_ (Rentel and Knight, 2004).

**Fig. 3.**
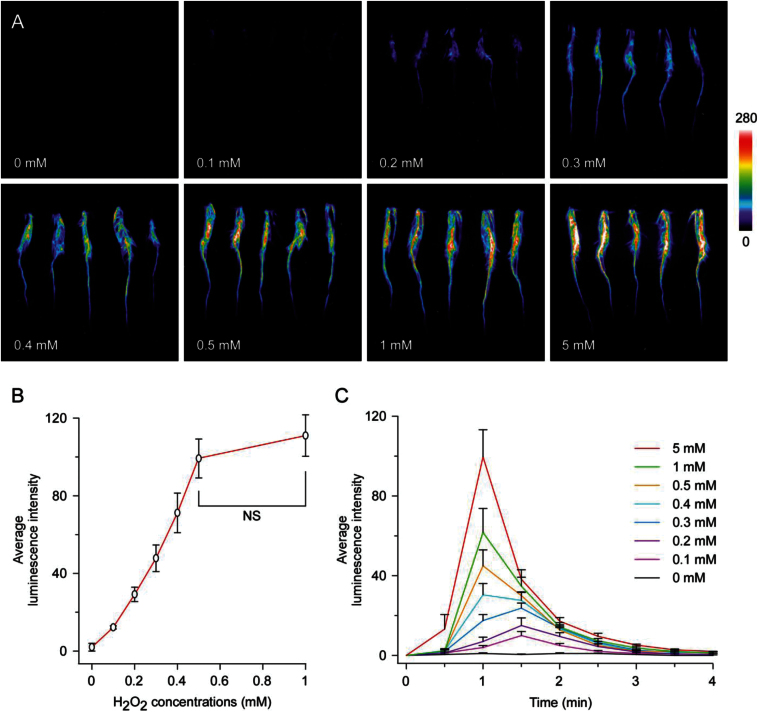
Aequorin-based luminescence in roots under H_2_O_2_ treatments. (A) Pseudocolour images of aequorin luminescence in roots treated with different concentrations of H_2_O_2_. The relationship between luminescence intensity and the pseudocolour images are scaled by a pseudocolour bar. (B) The line chart of luminescence signal intensity of every treatment. (C) The time courses of [Ca^2+^]_i_ changes induced by different concentrations of H_2_O_2_ in rice roots. Data for independent experiments are shown (mean±sd; *n*=10; NS, not significant *P*>0.05; Student’s *t*-test).

To investigate the H_2_O_2_ concentration-dependent increase of [Ca^2+^]_i_ in rice roots, we examined the aequorin-based luminescence signals and calculated the average luminescence intensity of rice roots in response to different concentrations of H_2_O_2_ treatments. Overall, the H_2_O_2_ response was quite similar to the NaCl response, when looking only at the concentration-dependent increase in luminescence (Fig. 3A). A concentration of 0.2mM H_2_O_2_ was able to induce clear luminescence signals, and the more H_2_O_2_ was applied, the stronger the luminescence signals would be (Fig. 3A). Detailed analysis showed that there was almost a linear relationship between luminescence signals and the H_2_O_2_ concentrations when the concentration of H_2_O_2_ was low (below 0.5mM). Higher concentrations of H_2_O_2_ (more than 0.5mM) were able to induce stronger luminescence signals but with a reduced rate of increase (Fig. 3B). Concentrations of H_2_O_2_ higher than 5mM had little additional effect on the luminescence changes (Supplementary Fig. S5).

Next, we investigated the time courses of increase in [Ca^2+^]_i_ induced by different concentrations of H_2_O_2_ in rice roots. H_2_O_2_ did not induce an immediate spike of [Ca^2+^]_i_ as NaCl did. Our results showed that within the first 30 s, only the highest concentration of H_2_O_2_ (5mM) was able to induce any luminescence signal, this is in contrast to NaCl which within 30 s was able to induce luminescence signals for all but the lowest concentration (0.1M) (Fig. 3C compared with Fig. 2C). Furthermore, the phase of [Ca^2+^]_i_ responses were also different among treatments with different concentrations of H_2_O_2_. Treatments with higher concentrations of H_2_O_2_ (more than 0.4mM) had a peak between 30 s and 1min, lower concentrations of H_2_O_2_ (below than 0.4mM) would delay the peak by about 30 s. Moreover, the spike of [Ca^2+^]_i_ induced by H_2_O_2_ did not decline to the basal level as quickly as NaCl. The luminescence signals declined gradually to the basal level within 3min (Fig. 3C). These results revealed that H_2_O_2_ induces a delayed [Ca^2+^]_i_ spike compared to NaCl.

### The different effects of Ca^2+^ inhibitors in NaCl- and H_2_O_2_-induced [Ca^2+^]_i_ responses and downstream gene expression

**Fig. 4.**
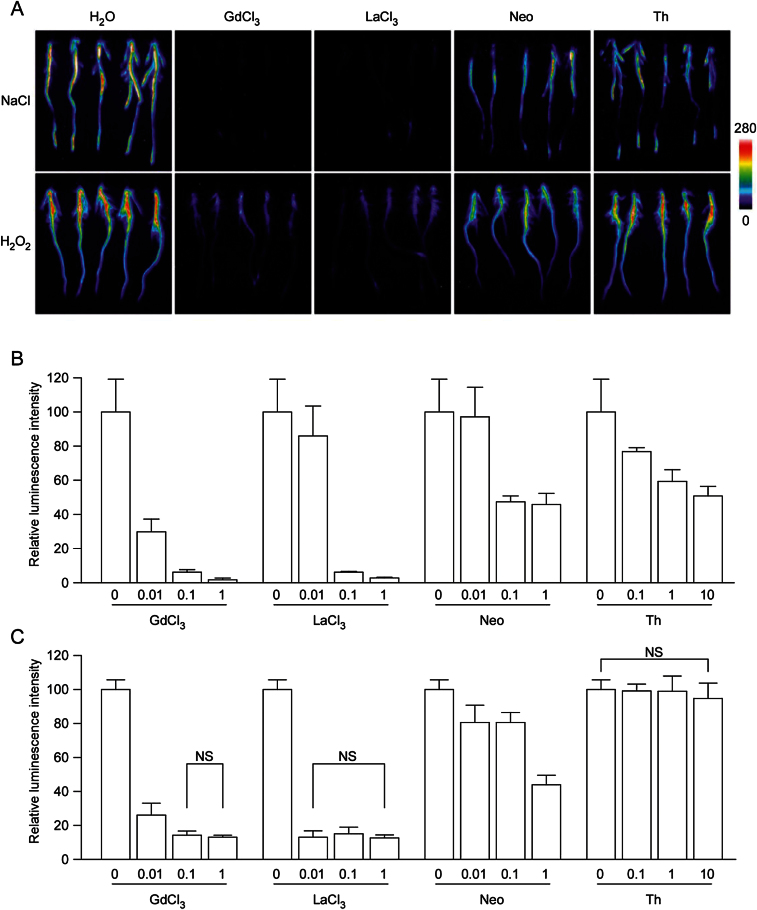
The effects of Ca^2+^ inhibitors in NaCl- and H_2_O_2_-induced [Ca^2+^]_i_ increases. (A) Treatment of Ca^2+^ inhibitors (1mM of GdCl_3_, LaCl_3_ and neomycin, 10 μM of thapsigargin) differentially reduced NaCl and H_2_O_2_ induced [Ca^2+^]_i_ changes. The relationship between luminescence intensity and the pseudocolour images are scaled by a pseudocolour bar. (B, C) The effects of different concentrations of Ca^2+^ inhibitors in NaCl (B) and H_2_O_2_ (C) induced [Ca^2+^]_i_ increases. The concentration gradient for GdCl_3_, LaCl_3_ and neomycin are 0.01mM, 0.1mM and 1mM; for thapsigargin it is 0.1 μM, 1 μM and 10 μM. Data for independent experiments are shown (mean±sd; *n*=10; NS, not significant *P*>0.05; Student’s *t*-test).

To further investigate the source of Ca^2+^ in NaCl- and H_2_O_2_-induced [Ca^2+^]_i_ responses, we tested GdCl_3-_, LaCl_3-_, neomycin- and thapsigargin-treated plants on the [Ca^2+^]_i_ increase in response to NaCl and H_2_O_2_ respectively. Gd^3+^ and La^3+^ are agonists of Ca^2+^, and they have been used as Ca^2+^ channel blockers to inhibit Ca^2+^ flux (Tracy *et al.*, 2008). In our experiment, GdCl_3_ and LaCl_3_ had similar inhibitory effects in NaCl- and H_2_O_2_-induced [Ca^2+^]_i_ increases respectively (Fig. 4). 1mM of GdCl_3_ and LaCl_3_ almost completely inhibited the [Ca^2+^]_i_ increase in response to NaCl, and inhibited about 90% of [Ca^2+^]_i_ increase in response to H_2_O_2_ (Fig. 4). Furthermore, significant dosage effects were observed except for LaCl_3_ in H_2_O_2_-induced [Ca^2+^]_i_ increase. Different concentrations of LaCl_3_ treatment had similar inhibitory effects in H_2_O_2_-induced [Ca^2+^]_i_ increase (Fig. 4B, C). Neomycin is an inhibitor of InsP_3_-stimulated Ca^2+^ release from internal stores (Munnik *et al.*, 1998). 0.01mM of neomycin treatment had no significant inhibitory effect, while 0.1mM and 1mM of neomycin treatment inhibited about 50% of [Ca^2+^]_i_ increase in response to NaCl. In the case of H_2_O_2_, 0.01mM and 0.1mM of neomycin treatment inhibited about 20% of [Ca^2+^]_i_ increase, while 1mM of neomycin treatment inhibited about 50% of [Ca^2+^]_i_ increase (Fig. 4B, C). Thapsigargin is an inhibitor of endoplasmic reticulum (ER) Ca^2+^-ATPases, and application of thapsigargin would empty the intracellular Ca^2+^ store in ER (Treiman *et al.*, 1998). In our experiment, thapsigargin significantly inhibited the NaCl-induced [Ca^2+^]_i_ increase, and a dosage effect for inhibition was observed. By contrast, thapsigargin had no significant effect on the H_2_O_2_-induced [Ca^2+^]_i_ increase (Fig. 4B, C). These results showed that the sources of Ca^2+^ in NaCl- and H_2_O_2_-induced [Ca^2+^]_i_ responses are different.

**Fig. 5.**
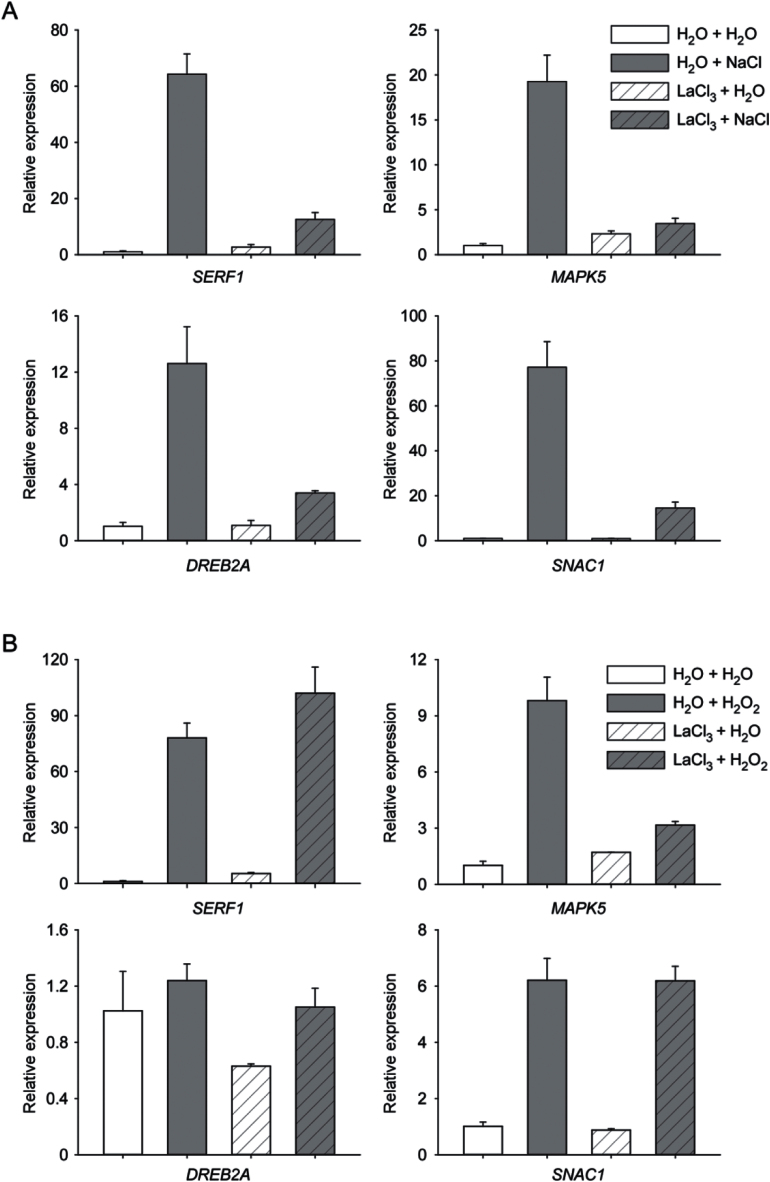
Relative expression levels of initial response genes induced by NaCl (A) and H_2_O_2_ (B). (A) H_2_O+H_2_O, untreated control. H_2_O+NaCl, NaCl treatment without pre-treatment. LaCl_3_+H_2_O, only LaCl_3_ pre-treatment, without NaCl treatment. LaCl_3_+NaCl, LaCl_3_ pre-treatment before NaCl treatment. (B) H_2_O+H_2_O, untreated control. H_2_O+H_2_O_2_, H_2_O_2_ treatment without pre-treatment. LaCl_3_+H_2_O, only LaCl_3_ pre-treatment, without H_2_O_2_ treatment. LaCl_3_+H_2_O_2_, LaCl_3_ pre-treatment before H_2_O_2_ treatment. Data for independent experiments are shown (mean±sd; *n*=3).

To evaluate the contribution of [Ca^2+^]_i_ responses in the NaCl- and H_2_O_2_-induced gene expression, the expression levels of *SERF1*, *MITOGEN-ACTIVATED PROTEIN KINASE5* (*MAPK5)*, *DEHYDRATION-RESPONSIVE ELEMENT BINDING2A (DREB2A)* and *STRESS-RESPONSIVE NAC1* (*SNAC1)*, which were reported to be induced by NaCl and H_2_O_2_ in rice, were analysed by quantitative RT-PCR (qRT-PCR) (Schmidt *et al.*, 2013). In our experiment, the expression levels of these genes were greatly increased by the NaCl treatment as reported previously (Schmidt *et al.*, 2013). After the pre-treatment of LaCl_3_, the induction by NaCl was seriously inhibited for all the genes examined, indicating a Ca^2+^-dependent manner of these inductions (Fig. 5A). On the other hand, H_2_O_2_ induced three of the four genes with *DREB2A* as the exception. Interestingly, only the expression of *MAPK5* showed Ca^2+^-dependent induction by H_2_O_2_. The expression of *SERF1* and *SNAC1* were induced by H_2_O_2_, however, pre-treatment of LaCl_3_ did not inhibit the induction of the expression by H_2_O_2_, indicating a Ca^2+^-independent manner of these inductions (Fig. 5B).

## Discussion

Since the transformation of aequorin in plants (Knight *et al.*, 1991), it has proved to be a useful tool for non-invasive investigation of Ca^2+^-mediated signalling in response to various stresses in whole seedlings (Zhu *et al.*, 2013). Furthermore, GAL4 transactivation of aequorin in enhancer trap lines enabled the testing of the stimulus- and cell-specific [Ca^2+^]_i_ signalling in specific tissues of *Arabidopsis* (Kiegle *et al.*, 2000; Martí *et al.*, 2013). In our experiment, the luminescence of aequorin was limited to rice roots, although the expression of apoaequorin was universal. It is unlikely that the leaf wax blocked the aequorin luminescence, because we detected chloroplast auto-fluorescence, which was emitted from the leaf cells (Fig. 1E). However, the leaf wax could have prevented the permeating of coelenterazine. To test this hypothesis, a surfactant was used to allow the coelenterazine to permeate through the leaf wax. After the addition of 0.01% surfactant, both strong luminescence signals in roots and weak luminescence signals in shoots were detected (Supplementary Fig. S1C). The luminescence signals in shoots were dotted, indicating insufficient permeation of coelenterazine. Although a higher concentration of surfactant (0.1%) increased the dotted luminescence signals in shoots, it greatly decreased the luminescence signals in roots, indicating the toxic effect of the surfactant to rice roots (Supplementary Fig. S1F). These results showed that our system is able to reflect the [Ca^2+^]_i_ level only in roots, not shoots.

In *Arabidopsis*, the discharging solution contains 10% ethanol, which is sufficient to permeate the exogenous Ca^2+^ and combine the remaining aequorin in the cell (Yuan *et al.*, 2014). In our experiment, we suggested a much higher concentration of ethanol (about 25%) to discharge all the aequorin. There are several differences in root radial structure between rice and *Arabidopsis*. From outside in, the epidermis, the ground tissue consisting of four tissues (exodermis, sclerenchyma cell layer, midcortex or mesodermis, and endodermis), and the central cylinder are present in a rice root, compared with single cell layers of epidermis, cortex, endodermis and the central cylinder in *Arabidopsis* (Dolan *et al.*, 1993; Rebouillat *et al.*, 2009). These additional cell layers in rice roots make it much thicker than that of *Arabidopsis*, and they form barriers that inhibit the permeation of exogenous Ca^2+^ in discharging solutions. In this research, we used luminescence intensity instead of real [Ca^2+^]_i_ in rice_._ Knight *et al.* (1996) described the equation to determine the Ca^2+^ concentration based on the luminescence intensity in *Arabidopsis*. However, the parameters in the equation can be variable among different species. Thus, for the accurate quantification of [Ca^2+^]_i_ in rice, a titration curve for analyzing the relationship between the Ca^2+^ concentration and luminescence intensity should be shown and real [Ca^2+^]_i_ should be estimated.

We investigated the [Ca^2+^]_i_ changes in response to various concentrations of salt stress, and found that it was sensitive within a very narrow range of NaCl concentrations (Fig. 2B). We observed that severe salt stress (more than 0.25M NaCl) did not increase [Ca^2+^]_i_ significantly compared with salt stress by 0.25M NaCl. Rice is a salt-sensitive crop and continued exposure to about 0.15M NaCl does not allow rice to complete its life cycle (Munns and Tester, 2008). In this view, there is no need for rice to evolve an energy wasting mechanism to respond to such a high concentration of NaCl. Alternatively, it is possible that severe salt stress (more than 0.25M NaCl) does increase [Ca^2+^]_i_ significantly. However, due to the saturation of our detection system, we failed to detect stronger luminescence signals when treated with high concentrations of NaCl. Moreover, we also observed that treatment with 0.1M NaCl (or less) did not induce an obvious increase of [Ca^2+^]_i,_ although it is sufficient to induce the expression of many salt response genes (Schmidt *et al.*, 2013). Our evidence suggests that the induction of these salt response genes are Ca^2+^ dependent (Fig. 5A); it is likely that low strength salt stress induces a very tiny [Ca^2+^]_i,_ response which is beyond the resolution of our detection system. It was reported that H_2_O_2_ triggered a biphasic [Ca^2+^]_i_ increase in *Arabidopsis* seedlings, and the second peak 5 to 20min after stress application was located exclusively in the root (Rentel and Knight, 2004). However, in our experiment, we detected the luminescence signals only in the first 3min and failed to detect additional signals even with the time extended to 45min (Supplementary Fig. S4). Considering the different environments rice and *Arabidopsis* roots are adapted to, rice may have evolved a different mechanism to cope with ROS stress.

We investigated the time courses of [Ca^2+^]_i_ changes in response to different concentrations of NaCl and H_2_O_2_ treatments in rice roots. Treatments of NaCl at different concentrations induced immediate [Ca^2+^]_i_ spikes within 30 s (Fig. 2C). In contrast, H_2_O_2_ did not induce any [Ca^2+^]_i_ increases within the first 30 s unless the concentration of H_2_O_2_ was high (Fig. 3C). In the view that the salt sensors are still unknown in plants, we propose that the salt sensors may be on the surface of the root and are closely coupled with Ca^2+^ channels. These salt sensors can respond to different concentrations of salt directly. On the other hand, it is likely that the effect of H_2_O_2_ on calcium signalling is less direct. The treatment of roots with H_2_O_2_ will in the first instance cause oxidation of the apoplast, and potentially it can affect calcium fluxes once it has been taken up by the cell through aquaporins, and thus a delay in response is observed. The H_2_O_2_- induced [Ca^2+^]_i_ spikes extended for several minutes (Fig. 3C), which also indicated a complicated and indirect way of H_2_O_2_-induced [Ca^2+^]_i_ response.

GdCl_3_ and LaCl_3_ are widely used to block Ca^2+^ channels (Tracy *et al.*, 2008). As expected, 1mM of GdCl_3_ and LaCl_3_ almost completely inhibit the [Ca^2+^]_i_ increase in response to NaCl. Interestingly, although 1mM of GdCl_3_ and LaCl_3_ were able to inhibit about 90% of [Ca^2+^]_i_ increase in response to H_2_O_2_, it cannot go further. There were no significant differences between 0.1mM and 1mM of blocker treatments, indicating a saturation of Ca^2+^ channels blocked by GdCl_3_ and LaCl_3_ (Fig. 4B, C). These results suggested that H_2_O_2_ is able to induce a [Ca^2+^]_i_ increase via different channels, and a small portion of these channels are GdCl_3_/LaCl_3_ insensitive. We also examined the source of Ca^2+^ in NaCl- and H_2_O_2_- induced [Ca^2+^]_i_ responses by treatment with neomycin, which is an inhibitor of InsP_3_-stimulated Ca^2+^ release from internal stores (Munnik *et al.*, 1998). Neomycin inhibited about 50% of [Ca^2+^]_i_ increase in response to both NaCl and H_2_O_2_. These results suggested that the [Ca^2+^]_i_ increases caused by NaCl and H_2_O_2_ treatments came from both internal and external stores of Ca^2+^. ER is an important internal Ca^2+^ store in the cell (Franzini-Armstrong, 1998). In our experiment, we used thapsigargin to empty the intracellular Ca^2+^ store in ER. As a result, NaCl induced [Ca^2+^]_i_ increase was significantly inhibited, indicating a participation of ER in the NaCl-induced [Ca^2+^]_i_ increase. Interestingly, thapsigargin had no significant effect on the H_2_O_2_-induced [Ca^2+^]_i_ increase (Fig. 4B, C). This suggested that another internal Ca^2+^ store, rather than ER, may participate in the H_2_O_2_-induced [Ca^2+^]_i_ increase.

**Fig. 6.**
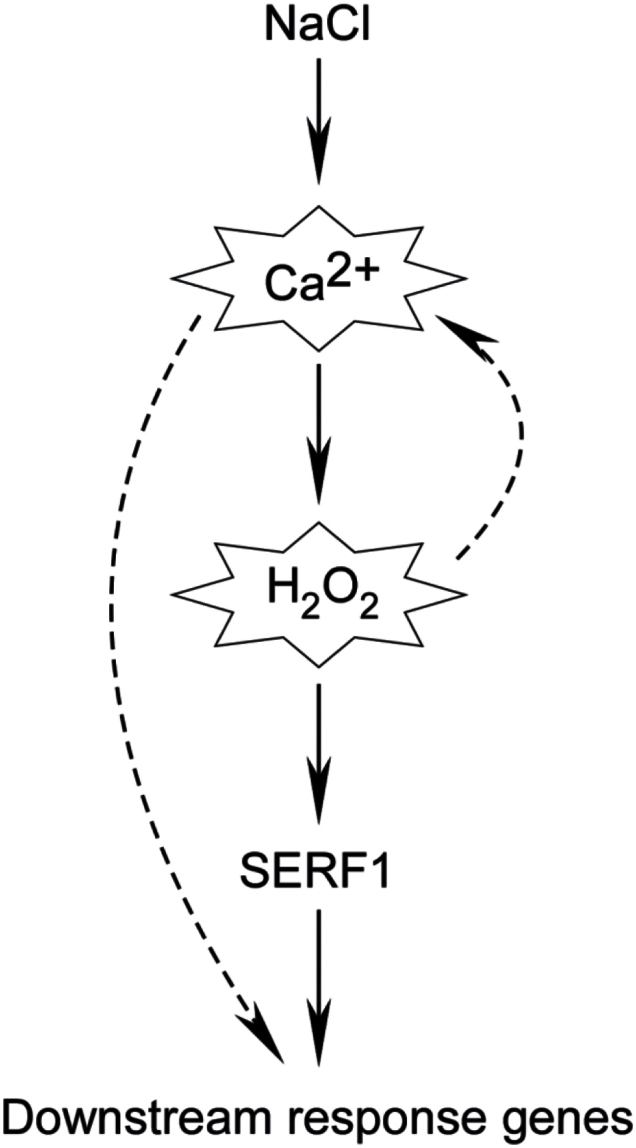
Proposed role of Ca^2+^ and H_2_O_2_ during the initial response to NaCl. NaCl as a primary signal induces a [Ca^2+^]_i_ spike, and the increase of [Ca^2+^]_i_ triggers a ROS burst. The increased concentration of H_2_O_2_ induces the expression of *SERF1* and downstream initial response genes (solid arrows). In addition, H_2_O_2_ also induces a [Ca^2+^]_i_ spike, and the increase of [Ca^2+^]_i_ is needed for the expression of some initial response gene(s) (dotted arrows).

Recently, the molecular processes controlling early stress perception and signalling have been explored and several genes have been identified as initial response genes in rice (Dai Yin *et al.*, 2005; Schmidt *et al.*, 2013). Among these genes, *SERF1* has a critical role and functions as a central hub in the ROS-dependent signalling during the initial response to salt stress in rice (Schmidt *et al.*, 2013). In our research, we examined NaCl- and H_2_O_2_- induced expression levels of *SERF1* and other initial response genes with or without LaCl_3_. As expected, *SERF1* and all the other initial response genes examined were induced by NaCl treatment. Furthermore, the induction of all these genes was seriously inhibited by the pre-treatment of LaCl_3_ (Fig. 5A). In the H_2_O_2_ treatment experiments, expression of the initial response genes *SERF1* and *SNAC1* were induced. However, the induction of these genes was not inhibited by the pre-treatment of LaCl_3_ (Fig. 5B). Considering the fact that a [Ca^2+^]_i_ spike is the first response to salt stress (Knight *et al.*, 1997), and salt-induced ROS accumulation is regulated by Ca^2+^ (Drerup *et al.*, 2013), we propose a Ca^2+^ and H_2_O_2_ mediated molecular signalling model for the initial response to NaCl in rice (Fig. 6). Salt stress immediately induces a [Ca^2+^]_i_ spike, and the increase of [Ca^2+^]_i_ triggers a ROS burst probably through the activation of NADPH oxidases. The increased H_2_O_2_ induces the expression of *SERF1* and downstream initial response genes. In addition, H_2_O_2_ also induces a [Ca^2+^]_i_ spike, and the increase of [Ca^2+^]_i_ is needed for the expression of some initial response gene(s), such as *MAPK5*. In our experiment, H_2_O_2_ did not induce the expression of *DREB2A* significantly as previously described (Schmidt *et al.*, 2013). We reasoned that 1h of H_2_O_2_ treatment may not be long enough to induce the expression of *DREB2A*. In the previous report, 3h of H_2_O_2_ treatment was able to induce the expression of *DREB2A*, while 30min of H_2_O_2_ treatment had no obvious effect (Schmidt *et al.*, 2013). Considering the fact that 1h of NaCl treatment is sufficient to induce the expression of *DREB2A* (Fig. 5A), a complex network rather than a linear structure may exist to regulate the expression of *DREB2A* (Kim *et al.*, 2011).

In summary, based on the aequorin luminescence, we established a calcium reporter line in rice. Using this, we tested the different [Ca^2+^]_i_ responses to NaCl and H_2_O_2_ in rice roots. Ca^2+^ inhibitor treatments revealed that both external and internal Ca^2+^ stores are involved in the [Ca^2+^]_i_ increases induced by NaCl and H_2_O_2_. Further research suggested that the internal Ca^2+^ store in ER participates in the NaCl-induced [Ca^2+^]_i_ increase, while another internal Ca^2+^ store, rather than ER may participate in the H_2_O_2_- induced [Ca^2+^]_i_ increase. In addition, we dissected the early salt-stress signalling cascade and presented a Ca^2+^- and H_2_O_2_-mediated molecular signalling model for the initial response to NaCl in rice.

## Supplementary data

Supplementary data can be found at *JXB* online.

Supplementary Fig. S1. Leaf wax prevents the permeating of coelenterazine.

Supplementary Fig. S2. The optimization of discharging solution for luminescence imaging in rice.

Supplementary Fig. S3. Treatment with high concentration of NaCl resulted in serious cell death in the rice roots.

Supplementary Figure S4 H_2_O_2_ does not induce a second peak of [Ca^2+^]_i_ response.Supplementary Fig. S4. H_2_O_2_ does not induce a second peak of [Ca^2+^]_i_ response.

Supplementary Fig. S5. High concentrations of H_2_O_2_ have little additional effect on the luminescence changes.

Supplementary Table S1. The sequences of primers used in this research.

Supplementary Data
