# Supplementary material for: Aequorin-based luminescence imaging reveals differential calcium signalling responses to salt and reactive oxygen species in rice roots
Source: J Exp Bot. 2015 Mar 9;66(9):2535–45. doi: 10.1093/jxb/erv043 (PMC4986864; doi:10.1093/jxb/erv043)
Supplement: Supplementary Data [file supp_66_9_2535__index.html]

Aequorin-based luminescence imaging reveals differential calcium signalling responses to salt and reactive oxygen species in rice roots — Aequorin-based luminescence imaging reveals differential calcium signalling responses to salt and reactive oxygen species in rice roots — Supplementary Data 

# Aequorin-based luminescence imaging reveals differential calcium signalling responses to salt and reactive oxygen species in rice roots

## Supplementary Data

Data files

**Files in this Data Supplement:**

- Supplementary Data - Supplementary Data
- Supplementary Data - Supplementary Data
