# Supplementary material for: Aequorin-based luminescence imaging reveals differential calcium signalling responses to salt and reactive oxygen species in rice roots
Source: J Exp Bot. 2015 Mar 9;66(9):2535–45. doi: 10.1093/jxb/erv043 (PMC4986864; doi:10.1093/jxb/erv043)
Supplement: Supplementary Data [file supp_erv043_jexbot144329_file001.pdf]

Aequorin-based luminescence imaging reveals differential calcium signaling responses to salt and reactive oxygen species in rice roots

Yanyan Zhang[1,4], Yifeng Wang[2,4], Jemma L. Taylor[3], Zhonghao Jiang[1], Shu Zhang[1], Fengling Mei[1], Yunrong Wu[2], Ping Wu[2], Jun Ni[1*]

[1] College of Life and Environmental Sciences, Hangzhou Normal University, Hangzhou, China
[2] State Key Laboratory of Plant Physiology and Biochemistry, College of Life Science, Zhejiang University, Hangzhou, China
[3] School of Life Sciences, Gibbet Hill Campus, University of Warwick, Coventry, United Kingdom
[4] These authors contributed equally to this work

Figure S1

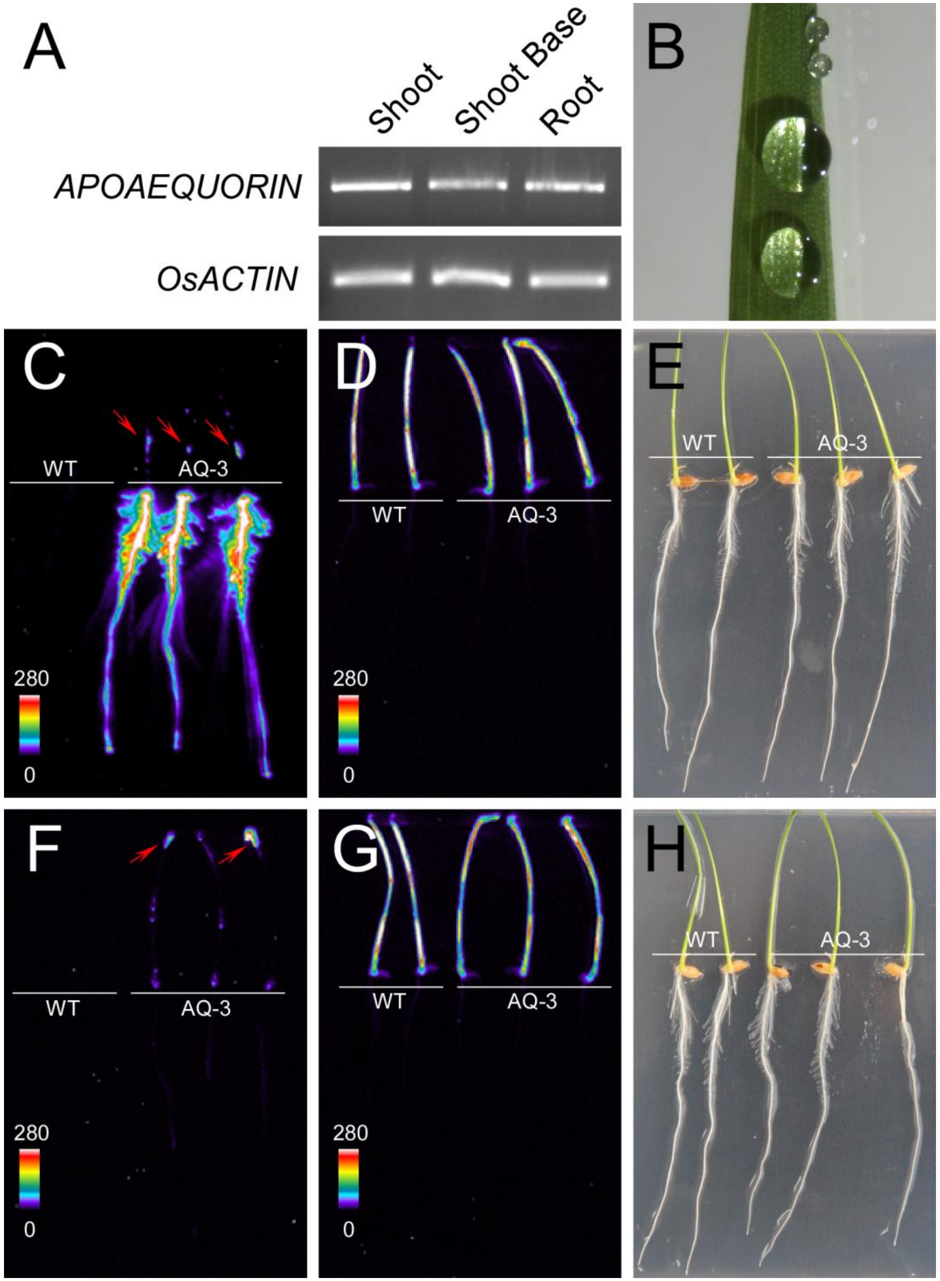
A
Shoot
Shoot Base
Root
APOAEQUORIN
OsACTIN
B
C
WT
AQ-3
280
0
D
WT
AQ-3
280
0
E
WT
AQ-3
F
WT
AQ-3
280
0
G
WT
AQ-3
280
0
H
WT
AQ-3

Figure S1 Leaf wax prevents the permeating of coelenterazine. (A) RT-PCR analysis of apoaequorin expression in different tissues. **(B)** The hydrophobicity of rice leaf wax. **(C-H)** Ca^{2+} treated seedlings showed luminescence in both roots and shoots after the addition of different concentrations of surfactant. **(C-E)** 0.01% of surfactant. **(F-H)** 0.1% of surfactant. **(C)** and **(F)** Pseudocolor images of aequorin luminescence in the whole plant. Arrows indicate dotted luminescence signals in shoots. **(D)** and **(G)** Pseudocolor images of chloroplast auto-fluorescence. **(E)** and **(H)** Bright-field images. The relationship between luminescence intensity and the pseudocolor images are scaled by pseudocolor bars.

Figure S2

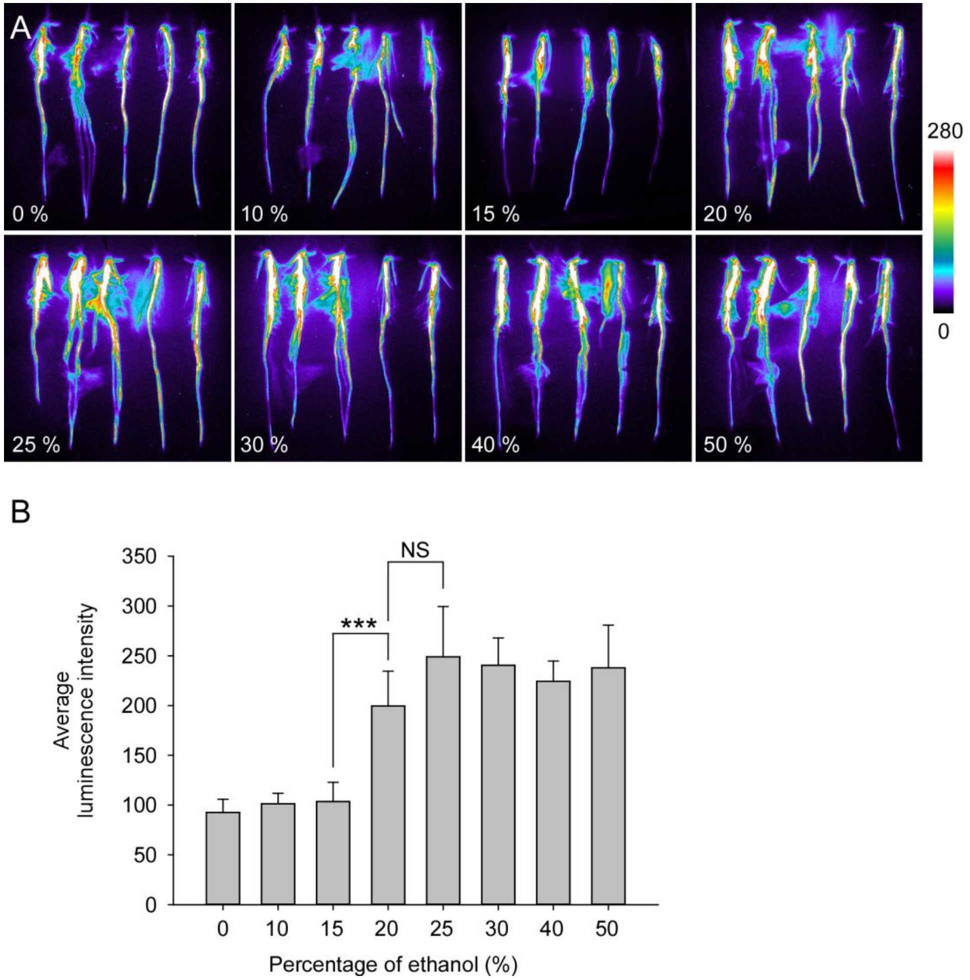

Figure S2 The optimization of discharging solution for luminescence imaging in rice. **(A)** Pseudocolor images of aequorin luminescence in roots treated by discharging solution containing different percentages of ethanol. The relationship between luminescence intensity and the pseudocolor images are scaled by a pseudocolor bar. **(B)** Luminescence signal intensity of every treatment. Data for independent experiments are shown (mean $\pm$ sd; n = 10; *** $P < 0.001$; NS, not significant $P > 0.05$; Student's *t*-test).

Figure S3

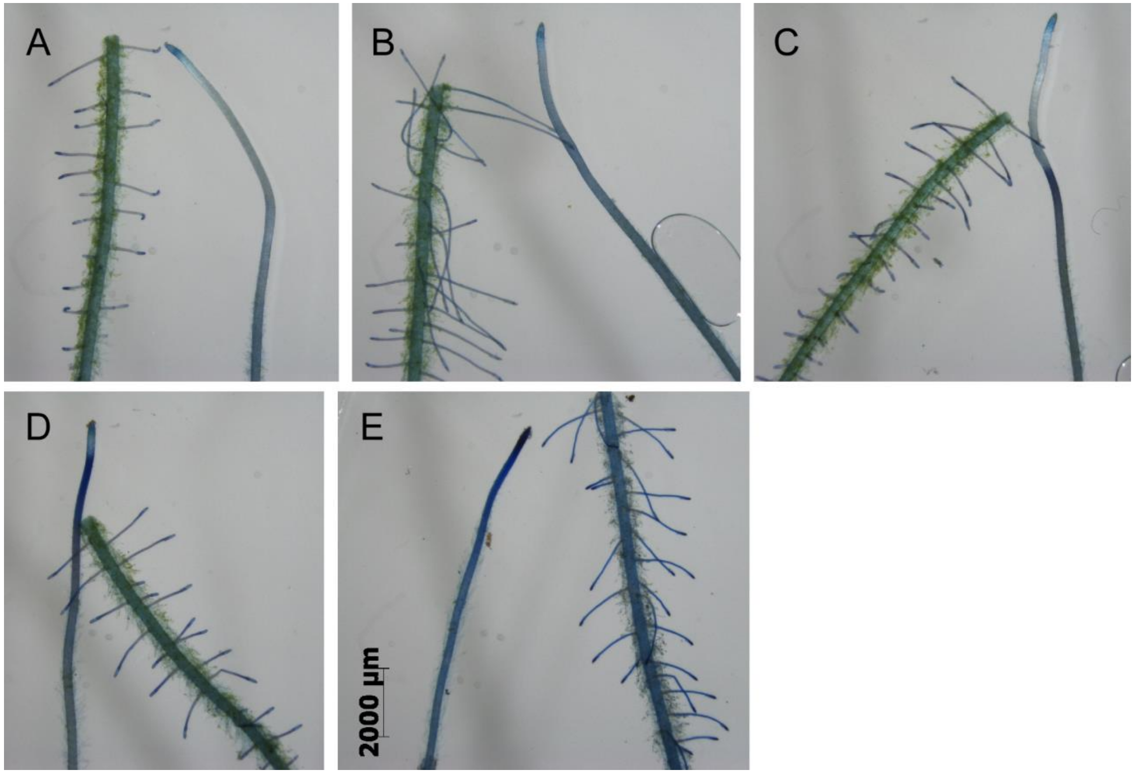

Figure S3 Treatment with high concentration of NaCl resulted in serious cell death in the rice roots. Roots were treated by different concentrations of NaCl solution for 30 sec and then stained by 1% Evans blue solution. **(A)** Untreated control, **(B)** Treated by 0.2 M NaCl, **(C)** Treated by 0.5 M NaCl, **(D)** Treated by 1 M NaCl, **(E)** Treated by 2 M NaCl. All the images used the same magnification.

Figure S4

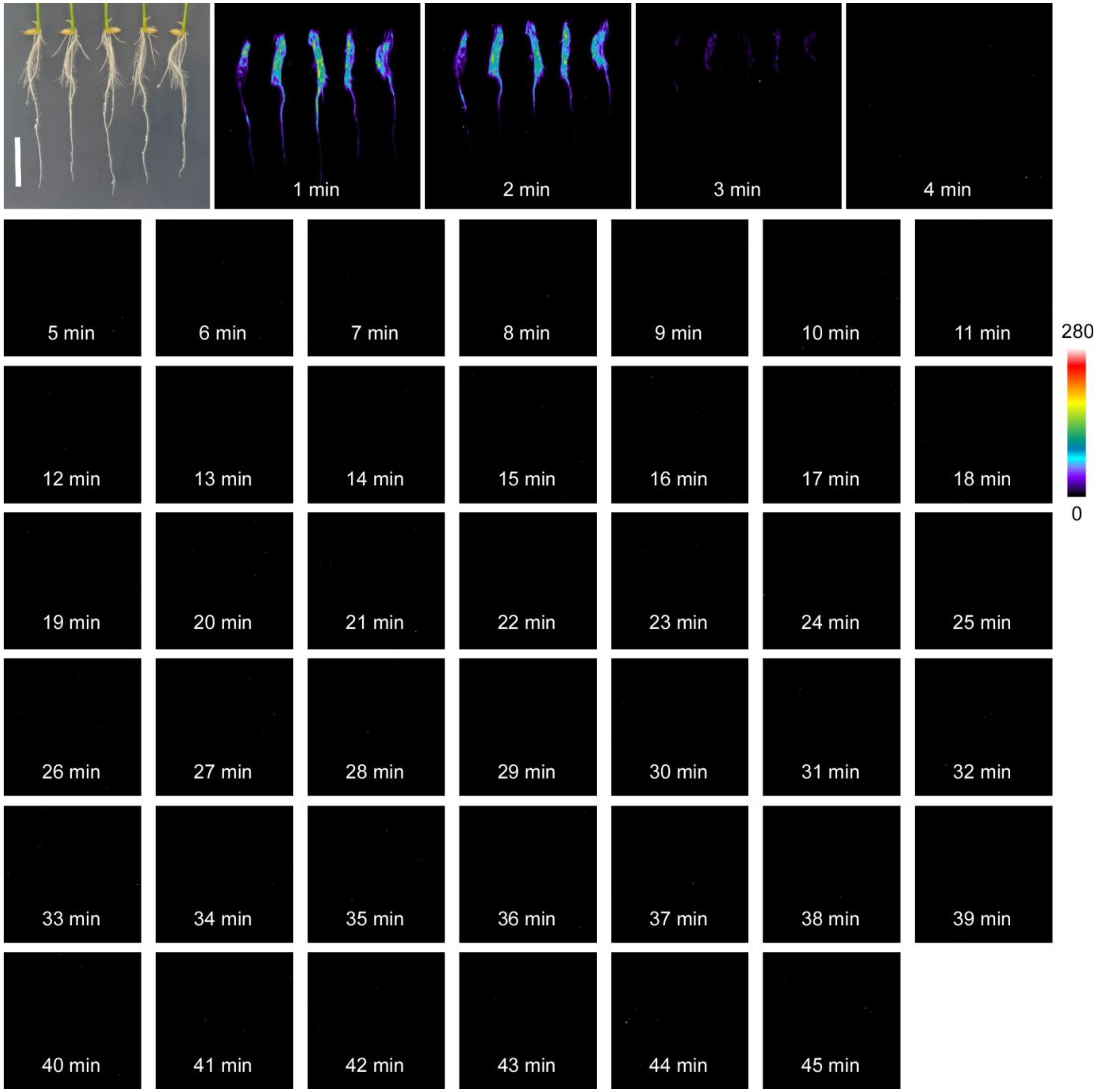
1 min
2 min
3 min
4 min
5 min
6 min
7 min
8 min
9 min
10 min
11 min
12 min
13 min
14 min
15 min
16 min
17 min
18 min
19 min
20 min
21 min
22 min
23 min
24 min
25 min
26 min
27 min
28 min
29 min
30 min
31 min
32 min
33 min
34 min
35 min
36 min
37 min
38 min
39 min
40 min
41 min
42 min
43 min
44 min
45 min
280
0

Figure S4 H_2O_2 does not induce a second peak of $[Ca^{2+}]_i$ response. Roots were treated by 1 mM H_2O_2 and the luminescence signals were collected 45 times at one min intervals after treatment with H_2O_2. The relationship between luminescence intensity and the pseudocolor images are scaled by a pseudocolor bar. The first picture is the same roots in bright-field, bar = 2 cm.

Figure S5

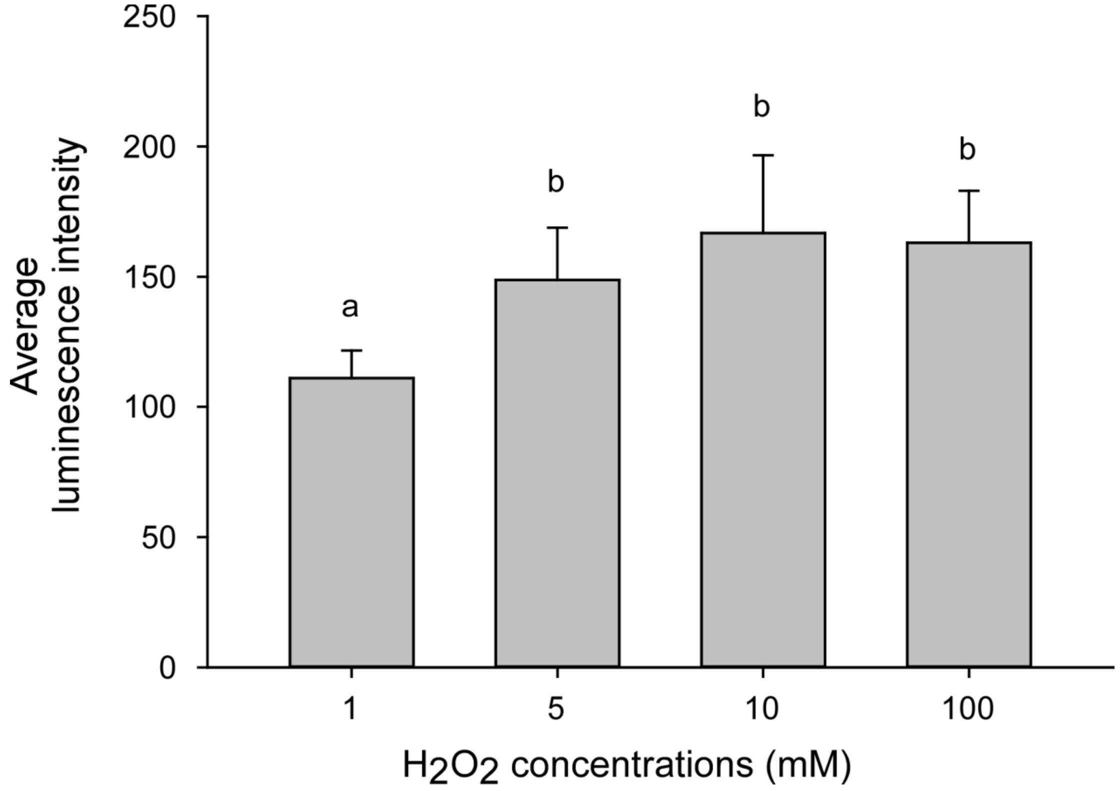

Figure S5 High concentrations of H_2O_2 have little additional effect on the luminescence changes. Roots were treated by 1 mM, 5 mM, 10 mM and 100 mM H_2O_2 respectively. The luminescence signals were compared by each other. Data for independent experiments are shown (mean ± sd; n = 10; Statistically distinct groups are marked by a and b; Student's *t*-test).
